# An Immune-Related Prognostic Signature for Predicting Clinical Outcomes and Immune Landscape in IDH-Mutant Lower-Grade Gliomas

**DOI:** 10.1155/2021/3766685

**Published:** 2021-12-18

**Authors:** Gang Xiao, Xuan Gao, Lifeng Li, Chao Liu, Zhiyuan Liu, Haiqin Peng, Xuefeng Xia, Xin Yi, Rongrong Zhou

**Affiliations:** ^1^Department of Radiation Oncology, Xiangya Hospital, Central South University, Changsha, Hunan 410008, China; ^2^State Key Laboratory of Microbial Resources, Institute of Microbiology, Chinese Academy of Sciences, Beijing 100101, China; ^3^GenePlus- Shenzhen Clinical Laboratory, Shenzhen 518122, China; ^4^Geneplus-Beijing, Beijing 102205, China; ^5^Xiangya Lung Cancer Center, Xiangya Hospital, Central South University, Changsha 410008, China

## Abstract

**Background:**

*IDH* mutation is the most common in diffuse LGGs, correlated with a favorable prognosis. However, the *IDH*-mutant LGGs patients with poor prognoses need to be identified, and the potential mechanism leading to a worse outcome and treatment options needs to be investigated.

**Methods:**

A six-gene immune-related prognostic signature in *IDH*-mutant LGGs was constructed based on two public datasets and univariate, multivariate, and LASSO Cox regression analysis. Patients were divided into low- and high-risk groups based on the median risk score in the training and validation sets. We analyzed enriched pathways and immune cell infiltration, applying the GSEA and the immune evaluation algorithms.

**Results:**

Stratification and multivariate Cox analysis unveiled that the six-gene signature was an independent prognostic factor. The signature (0.806/0.795/0.822) showed a remarkable prognostic performance, with 1-, 3-, and 5-year time-dependent AUC, higher than for grade (0.612/0.638/0.649) and 1p19q codeletion status (0.606/0.658/0.676). High-risk patients had higher infiltrating immune cells. However, the specific immune escape was observed in the high-risk group after immune activation, owing to increasing immunosuppressive cells, inhibitory cytokines, and immune checkpoint molecules. Moreover, a novel nomogram model was developed to evaluate the survival in *IDH*-mutant LGGs patients.

**Conclusion:**

The six-gene signature could be a promising prognostic biomarker, which is promising to promote individual therapy and improve the clinical outcomes of *IDH*-mutant gliomas. The study also refined the current classification system of *IDH*-mutant gliomas, classifying patients into two subtypes with distinct immunophenotypes and overall survival.

## 1. Introduction

Gliomas are the most common intracranial primary tumor, including nondiffuse gliomas and diffuse gliomas [1]. Diffuse gliomas were divided into lower-grade gliomas (grade II and grade III, LGGs) and glioblastoma (grade IV) by the 2016 World Health Organization classification based on the histological type [1, 2]. Gliomas patients harboring *IDH* mutations, including *IDH1* and *IDH2* mutation, have a better prognosis than the wild type [3]. Most of the LGGs with *IDH* wild type were molecularly and clinically analogous to primary glioblastoma [4]. *IDH* mutations often occur in LGGs patients with incidences of up to 75%, while the mutation frequency of *IDH1* is lower in glioblastoma (12%) [5, 6]. Among glioblastoma (GBM), *IDH* mutations mainly occur in secondary glioblastoma, progressing from *IDH*-mutant LGGs [1]. Except for gliomas, acute myeloid leukemia was the only cancer with a high incidence of *IDH1* mutations [7]. Based on *IDH* mutations along with the 1p/19q codeletion, tumor protein 53 (*TP53*) mutation, ATP-dependent X-linked helicase (*ATRX*) mutation, and telomerase reverse transcriptase (*TERT*) promoters mutation, gliomas can be classified into different groups with distinct pathogenesis and prognosis [1, 8, 9].

*IDH1* and *IDH2* genes encode isocitrate dehydrogenase 1 (IDH1) and isocitrate dehydrogenase 2 (IDH2) in gliomas. The mutation frequency of *IDH2* is far less than *IDH1*, and the two mutations rarely occur in the same patients [3, 10]. All the *IDH1* mutations are heterozygous missense mutations in codon 132. *IDH2* mutations are in codon 172, similar to the *IDH1* codon 132 [10, 11]. Both IDH1 and IDH2 are homodimers, catalyzing the oxidative decarboxylation of isocitrate into *α*-ketoglutarate and stabilizing cellular response to oxidative stress [5, 12, 13]. *IDH1* mutations can reduce enzymatic activity of the isocitrate and catalyze the formation of 2-hydroxyglutarate (2-HG) from *α*-ketoglutarate [14]. The accumulation of 2-HG is associated with brain tumorigenesis, but it also inhibits the proliferation of tumor cells. Besides, *IDH1* and *IDH2* mutations are early events in the development of gliomas [15]. Therefore, *IDH1* and *IDH2* mutations may be involved in the tumorigenesis of gliomas.

The standard of care for diffuse LGGs, including craniocerebral surgery, radiotherapy, and chemotherapy based on temozolomide or PCV regimen, failed to prevent tumor recurrence and progression [16]. Currently, immunotherapy plays an increasingly vital role in cancer treatment and is also being actively explored in gliomas [17]. Only glioblastoma patients were incorporated into clinical trials, but glioblastoma patients have not shown any survival benefit with nivolumab [18]. Recent studies have shown glioblastoma patients with methylated MGMT promoter and no baseline corticosteroid treated may benefit from immune checkpoint inhibitors (ICIs) [19]. To promote the application of immunotherapy in LGGs, the tumor immune microenvironment (TIME) needs to be investigated [20]. Studies revealed that *IDH* mutations were associated with immunosuppressive phenotypes [21, 22]. However, these studies did not explore the classification of *IDH*-mutant patients based on TIME and outcome. Moreover, few studies combined *IDH1* and *IDH2* mutations for further analysis.

Our study constructed a six-gene immune prognostic signature to predict overall survival in patients with *IDH*-mutant LGGs and divided these patients into subgroups with different outcomes and immunophenotypes. We identified that the signature was an independent prognostic factor, emphasizing the feasibility of the six-gene signature to be a clinical biomarker for *IDH*-mutant LGGs. Last, a predictive nomogram model integrating the signature and clinical factors was developed to predict the overall survival of *IDH*-mutant LGGs.

## 2. Materials and Methods

### 2.1. Data Collection

*IDH*-mutant LGGs patients (*n* = 800) from three cohorts were included in the study: CGGA_693 RNA sequencing (RNA-seq) cohort (*n* = 288), CGGA_325 RNA-seq cohort (*n* = 127), and TCGA RNA-seq cohort (*n* = 385). *IDH*-mutant GBM patients (*n* = 45) from the CGGA_693 cohort were included in the study. The corresponding molecular and clinical information of the two CGGA RNA-seq cohorts was downloaded from the CGGA database (http://www.cgga.org.cn/), of which the CGGA_693 cohort was regarded as the training cohort and the CGGA_325 cohort as the validation set [23–26]. Similarly, the TCGA RNA-seq cohort (https://portal.gdc.cancer.gov/) was downloaded by using TCGAbiolinks R package and was regarded as a validation cohort [27]. Univariate Cox regression analysis was applied to identify significantly overall survival-related genes (*P* < 0.05) with the CGGA_693 cohort and the TCGA RNA-seq cohort. Then, there were 253 overlapped protective genes (HR < 1) and 1864 risky genes (HR > 1) between these two cohorts. Finally, 105 immune-related prognostic genes were identified from the 2117 prognostic genes based on immune-related gene set from the Immunology Database and Analysis Portal (IMMPORT) database (https://www.immport.org/) [28]. Moreover, we performed GO and KEGG pathway analyses in David (http://david.abcc.ncifcrf.gov) for the functional annotation of the 105 candidate immune genes.

### 2.2. Construction of the Immune Prognostic Signature

Next, in the CGGA_693 cohort (the training set), the LASSO Cox regression analysis was done on the 105 immune-related prognostic genes to further reduce the number of immune genes by using the “glmnet” R package [29]. Then, 14 genes filtered by LASSO analysis were sent to multivariate Cox regression analysis to develop a six-gene immune-related prognostic signature in 288 *IDH*-mutant LGGs patients of the CGGA_693 cohort [30]. A six-gene-based risk score was calculated to evaluate each patient's risk by weighting the Cox regression coefficients. *IDH*-mutant LGGs in the CGGA_693 cohort were classified as either low or high risk by using the median risk score as a cutoff. Survival analysis was carried based on the Kaplan–Meier method and the log-rank test to evaluate the prognostic performance of the six-gene signature. The area under the curve (AUC) was calculated from the time-dependent receiver operating characteristic (ROC) curves to assess the sensitivity and specificity by using the “survivalROC” R package.

### 2.3. Gene Set Enrichment Analysis (GSEA)

To explore differences in the biological process between the high- and low-risk groups, GSEA was performed by using Java GSEA software with a threshold of nominal (NOM) *P* value <0.05 and a false discovery rate (FDR) <0.25. The c5.bp.v7.2.symbols.gmt and c2.cp.kegg.v7.2.symbols.gmt files were selected as the reference gene files.

### 2.4. Estimation of the Tumor Immune Microenvironment

The ESTIMATE algorithm was applied to access the infiltration of immune cells and stromal cells in tumor samples, which calculated the ESTIMATE score, immune score, stromal score, and tumor purity for each sample by the “estimate” R package [31]. Meanwhile, to explore the connection between the six-gene signature and the infiltration levels of immune cells, we comprehensively estimated the infiltration abundance of immune cells and stromal cells by applying four independent algorithms: xCell, TIMER, Cibersort, and MCP-counter [32–35]. Based on 28 innate and adaptive immune cells markers from Charoentong et al. study [36] and the markers of fibroblast and endothelial cells from the MCP-counter [35], the single sample gene set enrichment analysis (ssGSEA) algorithm was used to evaluate the infiltration abundance of 30 immune cells. Besides, we collected immunomodulators, including MHC molecules, immunostimulators, and immunoinhibitors, from Charoentong's study [36]. The abundance of exhausted T cells was estimated by utilizing ImmuCellAI (http://bioinfo.life.hust.edu.cn/ImmuCellAI) [37]. Meanwhile, 39 T-cell exhaustion-related genes were collected by reviewing the recent literature [38]. The genes list is shown in Table S1. We also collected gene signatures predicting immunotherapy and radiotherapy responses [39]. The enrichment scores of therapeutic signatures were calculated by applying the GSVA R package [40].

### 2.5. Estimation of Tumor Mutational Burden (TMB)

TCGAmutations R package was utilized to download somatic mutation of the TCGA LGGs cohort [41]. The somatic mutation and clinical information of the WESeq_286 cohort were downloaded from the CGGA database (http://www.cgga.org.cn/) [42, 43]. The TMB of each IDH-mutant LGGs patient with the TCGA and CGGA cohorts was estimated as previously described [44].

### 2.6. Construction of the Nomogram

To explore whether the six-gene signature could be an independent predictive factor of the overall survival for *IDH*-mutant LGGs patients, available molecular and clinical features (1p/19q codeletion status, MGMT promoter methylation, age, gender, grade, chemotherapy status, and radiotherapy status) were subjected to univariate and multivariate Cox regression analyses. Then, a nomogram was constructed based on the results of the multivariate Cox analysis by using the “rms” R package, which might predict the 1-, 3-, and 5-year overall survival of *IDH*-mutant LGGs patients. 1-, 3-, and 5-year OS calibration were conducted to evaluate the predictive accuracy of the nomogram. Moreover, we used the time-dependent ROC curves to compare the predictive performance of the six-gene signature with other independent prognostic markers by the survivalROC R package.

### 2.7. Statistical Analysis

All statistical analyses of our study were performed by using R 4.0.3. All reported *P* values were two-tailed in the study, and *P* < 0.05 was regarded as statistically significant. The median risk score was defined as a cutoff value throughout the study. The Mann–Whitney-Wilcoxon test was used for comparison of immune cell distribution, ESTIMATE score, and gene expression value.

## 3. Results

### 3.1. Screening Prognostic-Related Immune Genes in IDH-Mutant LGGs through Gene Expression

**Figure 1:**
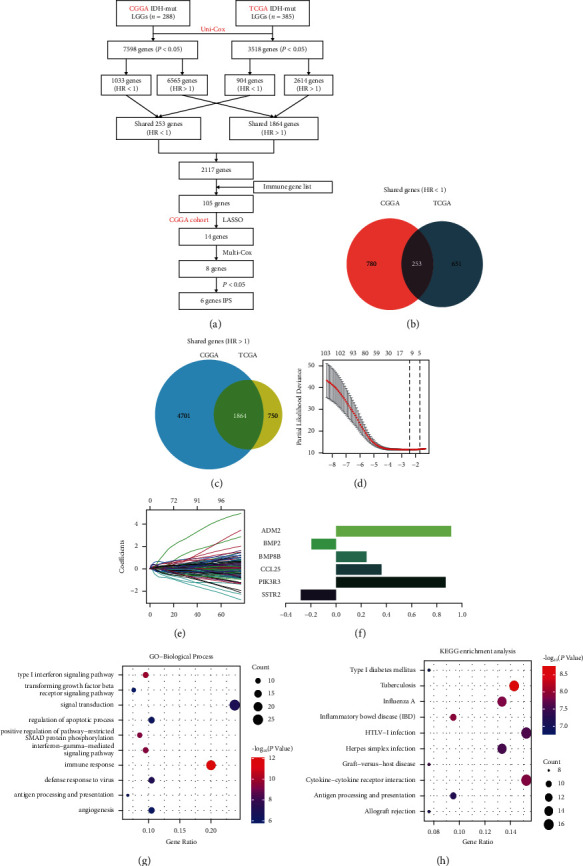
The study process of constructing a six-gene immune-related risk signature. (a) Flowchart of data analysis for screening immune-related prognostic genes. (b, c) The Venn diagram for the overlapped genes of HR > 1 and HR < 1 between the CGGA_693 and TCGA cohorts. (d, e) LASSO Cox regression identified 14 genes related to OS in the CGGA_693 cohort. (f) Coefficient values by multivariate Cox for six selected genes. (g, h) Functional annotation of 105 candidate immune genes using GO terms of biological processes and KEGG terms. CGGA: Chinese glioma genome atlas; TCGA: the cancer genome atlas; OS: overall survival; HR: hazard ratio; IPS: immune-related prognostic signature.

First, we screened 7598 and 3518 genes that were significantly correlated with overall survival from the *IDH*-mutant LGGs cohort of CGGA_693 (*n* = 288) and TCGA (*n* = 385) by applying univariate Cox regression analysis, respectively (Figure 1(a)). Then, we divided these genes into risky genes (HR > 1) and protective genes (HR < 1) based on hazard ratios (HR) values in the CGGA_693 and TCGA cohorts. The protective genes and risky genes were overlapped between the two cohorts, respectively (Figures 1(b) and 1(c)). Later, 2117 genes, including 1864 risky and 253 protective genes, were selected to overlap the immune gene sets downloaded from ImmPort Portal. Finally, we obtained 105 candidate immune genes, including 97 risky genes and 8 protective genes. The functional annotations of the 105 candidate immune genes were enriched in GO and KEGG terms, including signal transduction, immune response, antigen processing and presentation, and cytokine-cytokine receptor interaction (Figures 1(g) and 1(h)).

### 3.2. Construction of a Six-Gene Immune-Related Prognostic Signature for IDH-Mutant LGGs

**Figure 2:**
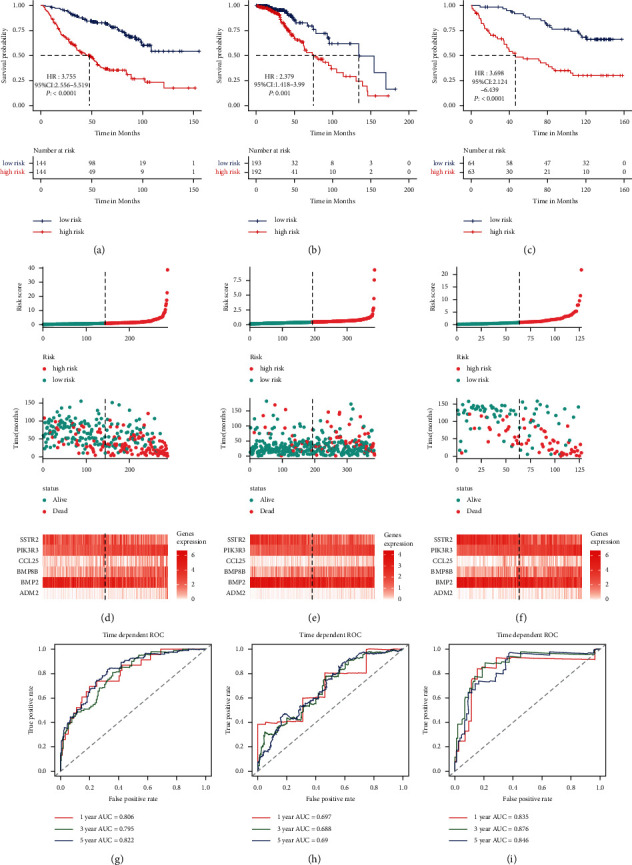
Validation of the six-gene risk signature. (a–c) Kaplan–Meier survival analysis for IDH-mutant LGG patients based on the risk signature in the CGGA_693, TCGA, and CGGA_325 cohorts. (d–f) Risk scores distribution, survival status of each patient, and expression profile of the six-gene signature in the three cohorts. (g–i) The time-dependent ROC curve of the six-gene signature in the three cohorts.

To develop an immune-related prognostic signature, 105 candidate immune genes were analyzed by the LASSO regression algorithm in the CGGA_693 cohort (Figures 1(a) and 1(e)). Then, we used multivariate Cox regression analysis for 14 genes from the LASSO regression algorithm to identify a risk signature. Finally, a six-gene risk signature, including two protective genes and four risky genes, was constructed in the CGGA_693 cohort (Figure 1(f)). The risk scores were calculated to predict prognostic value (risk score = 0.914 ∗ ADM2 + (−0.196) ∗ BMP2 + 0.242 ∗ BMP8B + 0.361 ∗ CCL25 + 0.869 ∗ PIK3R3 + (−0.280) ∗ SSTR2). The *IDH*-mutant LGGs patients in the CGGA_693 cohort were separated into low- or high-risk groups using the median risk score as the cutoff value. In *IDH*-mutant LGGs patients of the CGGA training cohort, the Kaplan–Meier analysis revealed that the high-risk group (*n* = 144) had a significantly worse OS than the low-risk group (*n* = 144) (median OS: 47.8 months versus not reach; HR for OS: 3.755 (2.556–5.519); *P* < 0.0001; Figure 2(a)). The gene expression profiles and risk score distribution are presented in Figure 2(d). The 1-, 3-, and 5-year AUC of the six-gene signature for OS were 0.806, 0.795, and 0.822 through the time-dependent ROC curve analysis in the CGGA_693 cohort, respectively, showing a moderate prognostic ability for OS of *IDH*-mutant LGGs (Figure 2(g)).

### 3.3. Validation of a Six-Gene Immune-Related Prognostic Signature for IDH-Mutant LGGs and IDH-Mutant GBM

To evaluate the predictive value of the signature, *IDH*-mutant LGGs patients from the TCGA (*n* = 385) and CGGA_325 cohorts (*n* = 127) were enrolled as the validation sets. The risk score of each *IDH*-mutant LGGs patient from the validation sets was calculated with the same formula. Using median value as the cutoff value, *IDH*-mutant LGGs patients were categorized as low- and high-risk patients. The high-risk group had a significantly shorter OS than the low-risk group in the TCGA cohort (median OS: 75.2 versus 134.3 months; HR for OS: 2.379 (1.418–3.990); *P*=0.001) and the CGGA_325 cohort (median OS: 46.3 months versus not reach; HR for OS: 3.698 (2.124–6.439); *P* < 0.0001), consistent with the CGGA_693 cohort results (Figures 2(b) and 2(c)). The gene expression profiles and risk score distribution were presented in Figures 2(e) and 2(f). The 1-, 3-, and 5-year AUC of the six-gene signature for OS were 0.697/0.688/0.690 and 0.835/0.876/0.846 through the time-dependent ROC curve analysis in the TCGA and CGGA_325 cohorts, respectively (Figure 2(h) and 2(i)). Therefore, the risk signature also indicated a promising prognostic ability for OS of *IDH*-mutant LGGs in validation sets. Moreover, we evaluated the predictive value of the six-gene signature in the *IDH*-mutant GBM cohort. The risk score of each *IDH*-mutant GBM patient from the CGGA_693 cohort (*n* = 45) was calculated similarly, respectively. We found that high-risk patients had a worse OS than low-risk patients in the *IDH*-mutant GBM cohort (median OS: 13.9 versus 33.6 months; HR for OS: 2.869 (1.351–6.091); *P*=0.0061; Supplementary Figure 1).

### 3.4. Predictive Role of the Six-Gene Immune-Related Signature with the Survival in Various Clinical Characteristics

**Figure 3:**
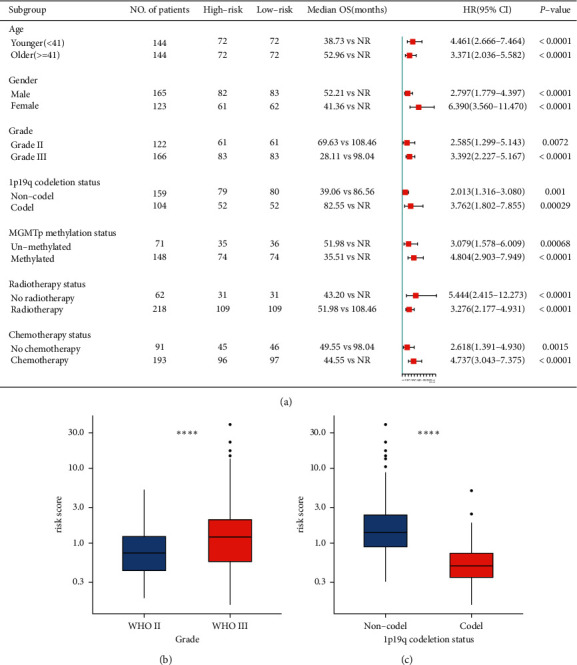
Stratification analysis based on clinicopathological and molecular information. (a) The forest plot for survival analysis of the risk signature in patients with female or male, younger or older, WHO grade II or WHO grade III, no chemotherapy or chemotherapy, no radiotherapy or radiotherapy, 1p19q noncodeletion or 1p19q codeletion, and unmethylated MGMT promoter or methylated MGMT promoter. The risk score was grouped by tumor grade (b) and 1p19q codeletion status (c).

To test the stability of the signature in different subgroups, we conducted stratification analyses by dividing the CGGA_693 cohort into different subgroups based on clinicopathological and molecular information (age, gender, WHO grade, radiotherapy status, chemotherapy status, 1p19q codeletion status, and MGMT promoter methylation status). As shown in Figure 3(a), the low-risk group had a significantly longer median OS than the high-risk group in patients with female or male, younger or older, WHO grade II or WHO grade III, no chemotherapy or chemotherapy, no radiotherapy or radiotherapy, 1p19q noncodeletion or 1p19q codeletion, and unmethylated MGMT promoter or methylated MGMT promoter. We also evaluated the correlation between clinicopathological/molecular parameters and risk scores. The value of risk score was higher in 1p19q noncodeletion and WHO grade III (*P* < 0.05, Figures 3(b) and 3(c)).

### 3.5. Immune Landscape of High- and Low-Risk Patients with IDH-Mutant LGGs

**Figure 4:**
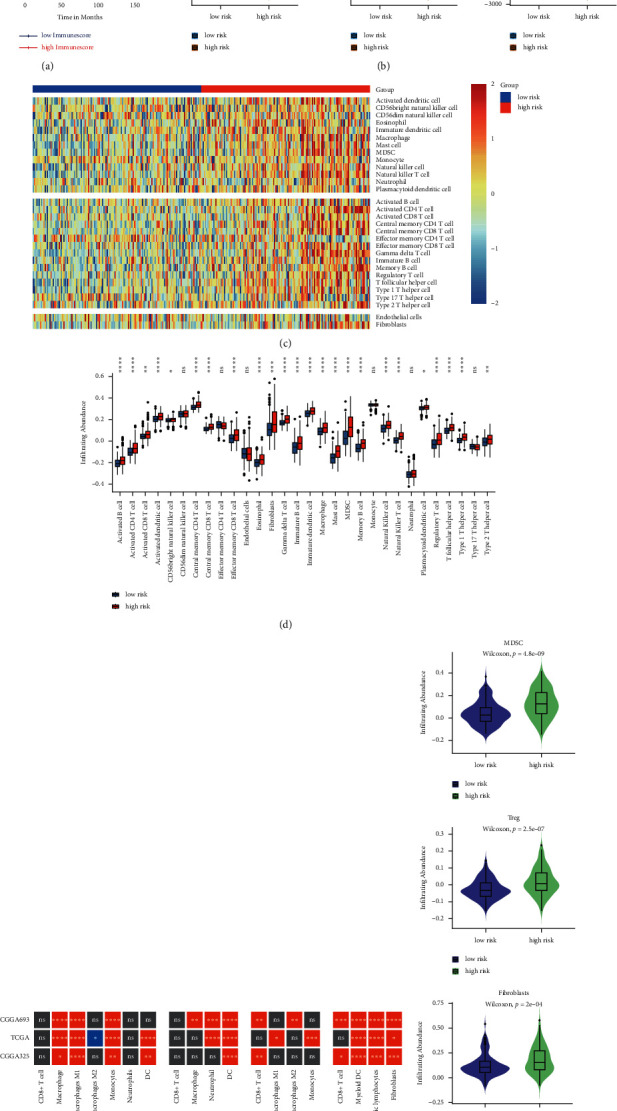
(a) Impact of the immune score on OS in IDH-mutant LGG based on Kaplan–Meier survival analysis. (b) The correlation between ESTIMATE score, immune score, stromal score, and risk score. The high-risk group showed a higher ESTIMATE score, immune score, and stromal score than the low-risk group. (c) The heatmap of 30 immune cells and stromal cells in the low- and high-risk groups. (d) The infiltrating difference of 30 immune cells and stromal cells between the two groups. (e) The infiltrating abundance of immune cells between two risk groups by applying four independent algorithms. (f) Comparison of the infiltrating abundance of MDSC, Treg, and fibroblasts between the low- and high-risk groups. The asterisks represent the statistical *P* value (ns: *P* > 0.05; ^*∗*^*P* < 0.05; ^*∗∗*^*P* < 0.01; ^*∗∗∗*^*P* < 0.001; ^*∗∗∗∗*^*P* < 0.0001).

We explored the distribution of 22 immune cells in the entire IDH-mutant LGG population. The most proportion of M2 macrophages and low proportion of CD8+ T cells were found in IDH-mutant LGGs patients, which may indicate an immunosuppressive tumor microenvironment (Figures S8). Further, we explored the differences in the immune infiltration characteristics between the low- and high-risk groups. We carried out ESTIMATE to investigate the immune characteristics and tumor purity between the low- and high-risk groups. Compared to the low-risk group, the high-risk group has a higher ESTIMATE score, immune score, and stromal score (*P* < 0.05; Figure 4(b)). In the *IDH*-mutant LGGs patients (*n* = 288) of the CGGA_693 cohort, the median immune score was used as the cutoff value to divide patients into high and low immune score groups. We found that the high immune score group had a worse outcome than the low immune score group (median OS: 67.3 versus 108.5 months; HR for OS: 1.809 (1.263–2.591); *P*=0.0012; Figure 4(a)).

To further explore the infiltration abundance of immune cells in LGGs patients with *IDH*-mutant, the ssGSEA algorithm was performed on the gene expression data of the *IDH*-mutant LGGs cohort (Figure 4(c)). We found that the high-risk group had higher infiltration levels of innate and adaptive immune cells, including CD8+ T cell, dendritic cell, and macrophage (Figure 4(d); Figure S4). Meanwhile, similar results were obtained by applying four independent immune algorithms to calculate the immune cells' infiltration level (Figure 4(e)). In contrast, a scarcity of innate immune cells and adaptive immune cells were shown in the low-risk group. However, we found that the abundance of immunosuppressive cells, including Treg and MDSC, and fibroblasts was higher in the high-risk group (Figure 4(f); Figure S4). Meanwhile, the abundance of exhausted T cells (Tex) and the expression level of T-cell exhaustion-related genes were higher in the high-risk group (Figure S5A). Altogether, we considered that the high-risk group could be an inflamed tumor immune microenvironment, but there could be T-cell exhaustion after immune activation. In contrast, the low-risk group with scarce immune cells could be related to the noninflamed tumor immune microenvironment.

### 3.6. Functional Annotation of the Six-Gene Risk Signature

**Figure 5:**
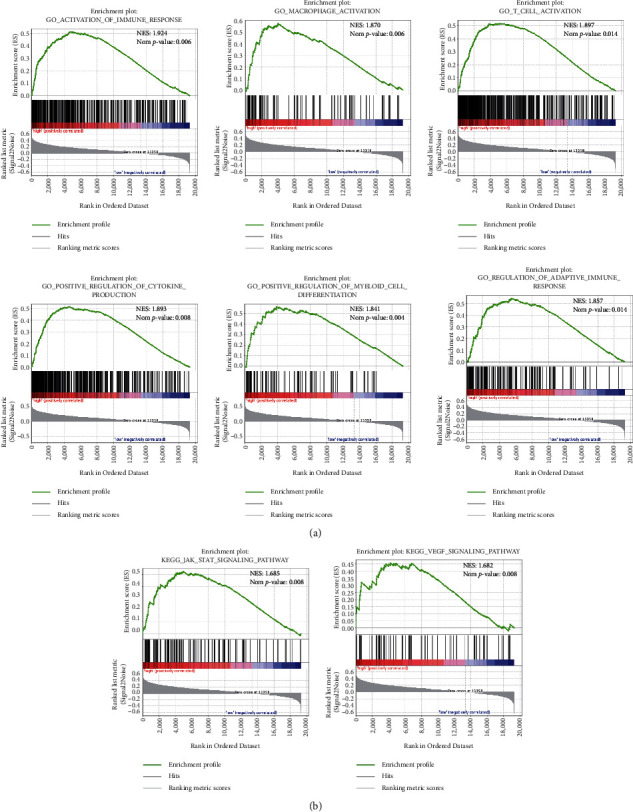
Functional difference between low- and high-risk groups in the CGGA_693 cohort. Gene set enrichment analysis (GSEA) for analyzing immune phenotype (a) and pathways (b) between low- and high-risk groups. NES: normalized enrichment score.

We conducted gene ontology (GO) enrichment analysis and the Kyoto Encyclopedia of Genes and Genomes (KEGG) pathways analysis based on GSEA analysis to gain insight into the different functions between low- and high-risk groups. We found that various immune-related biological processes (irBP), including activation of the immune response, macrophage activation, T-cell activation, positive regulation of cytokine production, positive regulation of myeloid cell differentiation, and adaptive immune response, were enriched in the high-risk group. In contrast, a few irBP were enriched in the low-risk group (Figure 5(a)). It suggested that the high-risk group may be associated with the enhanced immune phenotype.

**Figure 6:**
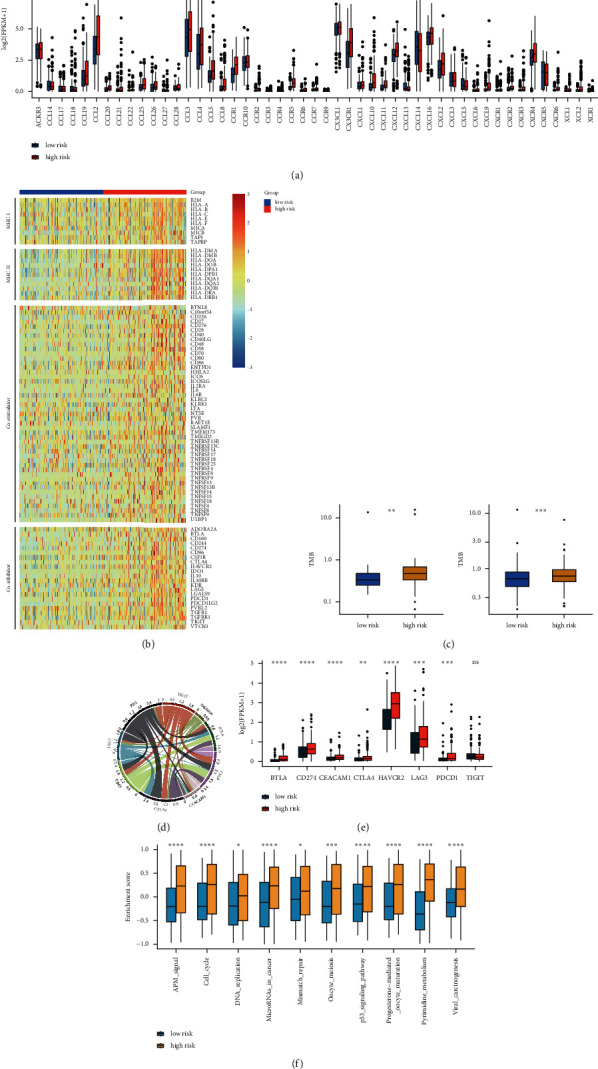
(a) The expression levels of chemokines and their receptor in the low- and high-risk groups. (b) The expression profile of immunomodulators molecule, including MHC molecule, immunostimulator, and immunoinhibitor, in the low- and high-risk groups. (c) Comparison of TMB in the low- and high-risk groups in the CGGA_693 and TCGA cohorts. (d) Chord diagram showing the correlation of the risk score with the expression of several critical immune checkpoint molecules. (e) Boxplot showing significantly different expression levels of immune checkpoint molecules between low- and high-risk groups. (f) Discordance in the enrichment scores of immunotherapy-predicted pathways between low- and high-risk groups in the CGGA_693 cohort.

To further explore the microenvironment characteristics, we compared the expression of cytokines between the low- and high-risk groups. The concentration of multiple chemokines, including CCL2, CCL4, CCL5, CCL19, CCL20, CXCL11, CXCL12, and CXCL13, and paired receptors, including CCR1, CCR2, CCR5, CCR6, CXCR3, and CXCR4, also was higher in the high-risk group. These chemokines and receptors were associated with the increase of effector tumor-infiltrating immune cells [39]. In particular, several crucial chemokines and receptors (CXCL9, CXCL10, and CXCR3), which can recruit CD8+ T cells into the tumor microenvironment [45], were upregulated in the high-risk group (Figure 6(a)). Besides, the high-risk group also exhibited increased expression levels in immunosuppressive chemokines, interleukins, and interferons. Several essential cytokines were also higher in the high-risk group, such as CCL5 (recruiting MDSC to tumor site), CCL22 (driving Treg recruitment into tumor site), and IL-10 (inhibiting cytokine synthesis) (Figure 6(a); Figure S2A) [46–48]. MHC I and MHC II expression were significantly higher in the high-risk group, showing a more robust antigen presentation capacity (Figure 6(b); Figure S6A). The high-risk group also exhibited higher expression of immunostimulators (Figure S6B). To explore the immunogenicity of *IDH*-mutant LGGs, we evaluated TMB based on CGGA and TGCA somatic mutation data of *IDH*-mutant LGGs. The TMB was significantly higher in the high-risk group, implying that the high-risk patients owned higher immunogenicity than low-risk patients (Figure 6(c)). Overall, we speculated that the low-risk group might show an intrinsic immune escape. Finally, we evaluated the expression levels of the immune checkpoints in the two groups, including PD-1, PD-L1, CTLA4, TIM-3, TIGIT, BTLA, and LAG-3. We found that the high-risk group had significantly higher PD-1, PD-L1, CTLA-4, TIM-3, BTLA, and LAG-3 expression levels than the low-risk group (*P* < 0.05; Figures 6(d) and 6(e); Figure S6C). Therefore, the high-risk group could evade the immune elimination after immune activation, owing to its high expression of immune checkpoints. The above results indicated that high-risk patients might benefit from ICIs. To further explore the feasibility of immunotherapy, we collected several immunotherapy-predicted pathways. The high-risk group had higher enrichment scores of immunotherapy-predicted pathways than the low-risk group (Figure 6(f)).

Furthermore, KEGG pathway analysis showed enriched pathways in the high-risk group, including the JAK-STAT signaling pathway and VEGF signaling pathway (Figure 5(b)). Besides, the high-risk group had higher expression levels of VEGFA and VEGFC than the low-risk group (Figure S2B). The high-risk group also had higher enrichment scores for predicting radiotherapy response pathways (Figure S3).

### 3.7. Six-Gene Immune Signature Is an Independent Prognostic Factor for IDH-Mutant LGGs

**Figure 7:**
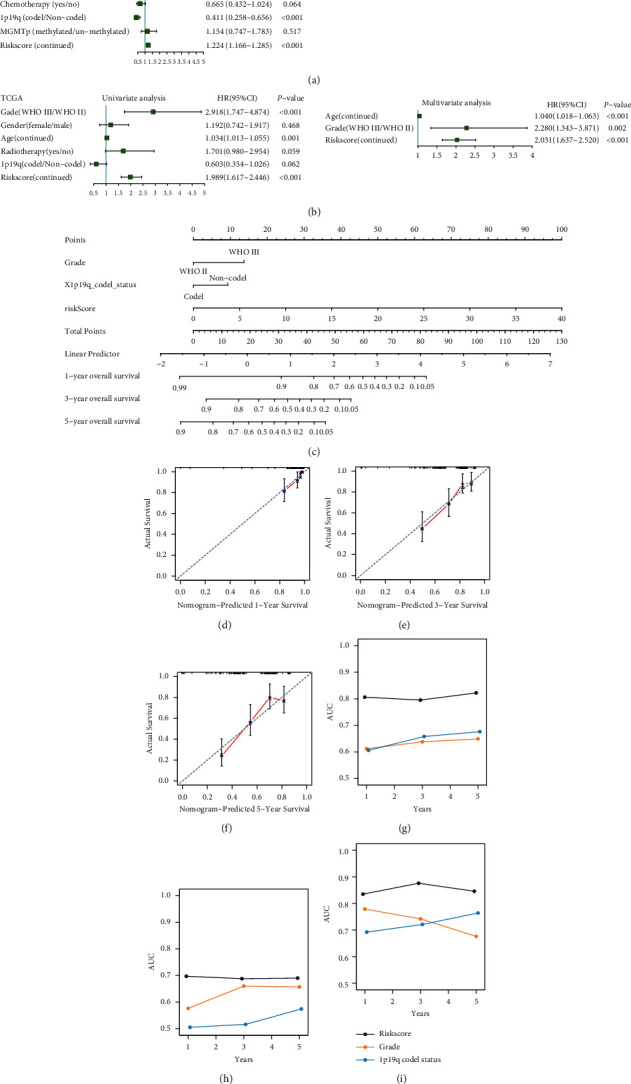
Constructing a nomogram model integrating the six-gene risk signature and clinical factors. (a, b) Univariate and multivariate Cox analyses showed that the risk signature was significantly correlated with OS in both CGGA_693 and TCGA cohorts. (c) Nomogram model for predicting the 1-, 3-, and 5-year survival in IDH-mutant LGGs. (d–f) Calibration curve of the nomogram for predicting the probability of OS at 1, 3, and 5 years. (g–i) The time-dependent AUC value of the six-gene signature and other clinical factors in the three independent cohorts.

We conducted the univariate and multivariate Cox regression analyses on risk score and the clinical/molecular features to identify whether this signature is an independent prognostic factor. Univariate Cox regression analysis showed that 1p19q codeletion status was significantly correlated with better survival, and grade and risk score were significantly correlated with worse survival in the CGGA_693 cohort (*P* < 0.05; Figure 7(a)). After adjusting for the available clinicopathological factors, multivariate Cox regression analysis indicated that the six-gene signature was an independent prognostic factor (HR for OS: 1.178, 95%CI: 1.119–1.240, *P* < 0.001; Figure 7(a)). Consistently, the six-gene signature was an independent prognostic indicator for OS, validated in the TCGA cohort (multivariate Cox: HR for OS: 2.031, 95%CI: 1.637–2.520, *P* < 0.001; Figure 7(b)) and the CGGA_325 cohort (multivariate Cox: HR for OS: 1.087, 95% CI: 1.002–1.178, *P* < 0.044; Figure S7). Therefore, the six-gene immune-related signature was an independent prognostic factor.

### 3.8. Nomogram Analysis

To provide a tool predicting the overall survival of *IDH*-mutant LGGs for the oncologist, we conducted a nomogram analysis integrating the risk signature, tumor grade, and 1p19q codeletion status (Figure 7(c)). To validate the accuracy of the nomogram, we carried calibration curves and time-dependent ROC analysis. The calibration curves of probabilities for 1-, 3-, and 5-year OS unveiled excellent agreement between actual and predicted survival (Figures 7(d)–7(f)). The time-dependent ROC curves were plotted to compare the predictive performance of the six-gene risk signature with the existing independent indicators. The six-gene risk signature (0.806/0.795/0.822) had higher 1-, 3-, and 5-year time-dependent AUC than grade (0.612/0.638/0.649) and 1p19q codeletion status (0.606/0.658/0.676), indicating that the six-gene risk signature had a better survival prediction performance (Figures 7(g)–7(i)).

## 4. Discussion

*IDH* mutation is the most stable in gliomas, which persists in primary, progressive, and recurrent gliomas [49, 50]. Previous studies suggested that *IDH1* mutation could be an essential contributor to cause the better survival of *IDH*-mutant glioma than the *IDH* wild type [51, 52]. However, the outcome and tumor immune microenvironment are also significantly discordant between different *IDH*-mutant gliomas patients. Therefore, gliomas patients with *IDH* mutation need to be further classified, identifying gliomas patients with poor prognoses to develop alternative treatment options. Unlike previous studies that only included *IDH1* mutations, we simultaneously enrolled LGGs with *IDH1* and *IDH2* mutations. Due to the specific prognosis and tumor microenvironment of *IDH-* mutant LGGs patients, we identified a six-gene immune-related prognostic signature in *IDH*-mutant LGGs. Our study data indicated that the high-risk group had worse survival and higher immune infiltration than the low-risk group, validated by TCGA and CGGA databases. Furthermore, our six-gene signature was identified as an independent prognostic factor in *IDH*-mutant LGGs, independent of known prognostic factors (tumor grade and 1p19q codeletion status). 1p19q codeletion status is a significant prognostic biomarker in LGGs, but many previous similar studies did not include the factor in analyses [53]. Meanwhile, we constructed a nomogram model based on the six-gene signature and other independent prognostic factors, including 1p19q codeletion status and tumor grade, to assist clinicians in predicting the survival of *IDH*-mutant LGGs patients. Moreover, our signature has a higher prognostic value than the existing clinical and molecular factors by comparing the AUC value, which is promising to be a biomarker of prognosis and immune status.

Our risk signature enrolled six immune-related genes, including *ADM2*, *BMP2*, *BMP8B*, *CCL25*, *PIK3R3*, and *SSTR2*. These genes play an essential role in tumor immune response, promising to be novel targets in cancer immunotherapy. *Adrenomedullin 2(ADM2)* gene encodes ADM2 protein, which is a member of the calcitonin gene-related peptide (CGRP) superfamily [54]. The biological functions of ADM2 are similar to Adrenomedullin [55]. There is evidence suggesting that ADM2 can affect macrophage polarization [56]. The *ADM2* expression was positively correlated with the malignancy grade in gliomas. As expected, GBM had the highest expression of *ADM2*. The ADM2 promoted GBM cell proliferation by activating ERK1/2 phosphorylation [57]. BMP2 and BMP8 are ligands of the transforming growth factor-*β* (TGF-*β*) family [58]. BMP2 could be a potentially immunomodulatory growth factor [59]. Previous studies suggested that endogenous BMP2 inhibited T-cell differentiation in the thymus [60, 61]. BMP2 promotes the differentiation and apoptosis of GBM cells. In addition, BMP2 raises GBM responsiveness to TMZ by inhibiting the hypoxia-inducible factor 1*α* (HIF-1*α*)/MGMT axis [62]. Therefore, the BMP2 was considered as a potential therapeutic target for GBM. The CCL25 is a CC motif chemokine ligand with just one receptor: CCR9 [63]. CCL25 may induce the infiltration of CD8+ T cells exhibiting CCR9 expression in tumors, showing an anticancer effect [64]. CCL25 can also recruit MDSC into the tumor microenvironment [65]. Phosphoinositide-3-kinase regulatory subunit 3 (PIK3R3), also known as p55GAMMA, is a member of the phosphatidylinositol 3-kinase (PI3K) family [66]. Previous studies confirmed that PIK3R3 could regulate the AKT/mTOR pathway, promoting cancer progression [67]. In glioma, overexpression of PIK3R3 can support the growth of GBM cells by engaging IGF2 signaling in vitro, indicating that PIK3R3 is an oncogene in glioma [68]. Somatostatin receptor 2 (SSTR2) is the family of G protein-coupled receptors and is widely expressed in solid tumors [69, 70]. SSTR2 is also expressed in inflammatory and lymphocytes cells, regulating the proliferative and secretory responses of these cells [69]. SSTR2 could mediate the cytostatic effects of somatostatin by activating phosphotyrosine phosphatase PTPeta and inhibiting ERK1/2 activity in C6 glioma cells [71]. Anaplastic oligodendrogliomas had a high expression level of SSTR2A, associated with a better outcome [72]. Therefore, SSTR2A might serve as a biomarker and target for gliomas.

The stability of prognostic models is crucial, deciding whether the model can be applied to a broader population. In our study, our six-gene risk signature is effective by identifying and validating in three independent cohorts containing the Chinese population and TCGA population. Moreover, our six-gene risk signature showed a better survival prediction performance in three cohorts with higher 1-, 3-, and 5-year time-dependent AUC. In general, our risk signature is stable and reliable.

Besides *IDH*-mutant LGGs, we explored the prognostic value of our six-gene signature in GBM with mutated *IDH*. We found that the six-gene signature was equally applicable for *IDH*-mutant GBM. In GBM patients with mutated *IDH*, the high-risk patients had a worse survival than the low-risk patients (*P* < 0.01). It indicated that the gene expression patterns were similar between different tumor grades of *IDH*-mutant gliomas. Meanwhile, our result also validated the previous viewpoint that most *IDH*-mutant GBM were secondary glioblastomas, which may evolve from *IDH*-mutant LGGs [49]. In the new WHO 2021 classification, as expected, all *IDH*-mutant diffuse astrocytic tumors are regarded as one type: Astrocytoma, *IDH*-mutant. In other words, the *IDH*-mutant GBM also is considered *IDH*-mutant astrocytoma. Therefore, our risk signature could be applied in all *IDH*-mutant gliomas.

A comprehensive understanding of the tumor immune microenvironment (TIME) in *IDH*-mutant LGGs can boost the comprehension of the mechanism of immunotherapy. First, we explored the overall immune cell infiltration status of the entire *IDH*-mutant LGGs population. We found that macrophages, particularly the M2 subtype, account for the most significant proportion in *IDH*-mutant LGGs patients, consistent with other reports that both microglia and macrophages are enriched in adult gliomas [73, 74]. Moreover, R-2-HG induces glioma-associated macrophages with suppressive phenotype in *IDH*-mutant gliomas [75]. CD8+ T cells had a paucity of infiltration levels in these patients, which may be since *IDH1* mutation suppressed the accumulation and activity of CD8+ T cells by the accumulation of 2-HG [22, 52, 76]. Overall, *IDH*-mutant LGGs showed a relatively suppressive immune phenotype [77]. Furthermore, the relationship between our risk signature and immune cell infiltration was investigated to uncover the status of TIME with low- and high-risk patients in *IDH*-mutant LGGs. Overall, the high-risk patients had a higher immune infiltration than the low-risk patients, including innate immune cells and adaptive immune cells. Contrary to our result, immune infiltration is positively correlated with overall survival in many types of tumors, including lung adenocarcinoma, colon cancer, melanoma, and head and neck squamous cell cancer [78–81]. There was a possible explanation that the tumor microenvironment of high-risk patients was mostly infiltrated with suppressive immune cells [51, 82]. Moreover, the high-risk group has a higher antigen presentation capacity (MHC molecule), immunogenicity (TMB), and immunostimulators levels (immunostimulatory molecule). However, we observed the specific immune escape mechanisms of the high-risk group based on the cancer Immunoediting hypothesis [83]. More immunosuppressive cells (MDSC, Treg, and fibroblast), immunosuppressive cytokines (CCL5, CCL22, and IL-10), and immune checkpoint molecules (PD-1, PD-L1, CTLA-4, TIM-3, LAG-3, and BTLA) were accumulated in the high-risk group, which could evade immune recognition and clearance after immune activation. Previous studies also unveiled that CD8+ T cells could upregulate the expression of PD-L1 and IDO and promote the recruitment of Tregs in the tumor microenvironment [48]. The high-risk group had more exhausted T cells and higher expression of T-cell exhaustion-related genes (including checkpoint molecular), implying that T cells lost polyfunctionality and renewal capacity in these patients. Thus, we speculated that the worse survival of high-risk patients could be partly owing to the specific immune escape and T-cell exhaustion in the tumor microenvironment.

Currently, the standard treatment of LGGs is inadequate, which is challenging to prevent glioma recurrence and progression. In our study, the high-risk group has a poor prognosis, showing an imperative need to develop alternative treatment options. Accordingly, we explored several treatment options that may serve high-risk patients. Firstly, given the increased expression level of immune checkpoint and infiltrating abundance of Tex in the high-risk group, immune checkpoint inhibitors (ICIs) could be efficacious to reverse T-cell exhaustion and specific immune escape. *IDH*-mutant LGGs patients in the high-risk group are more likely to benefit from ICIs. The increasing enrichment scores of immunotherapy-predicted pathways were also observed in the high-risk group, further showing the prospect of ICIs in these patients. Secondly, patients treated with radiotherapy (*n* = 114) had a longer median OS than those untreated (*n* = 24) in the high-risk group (median OS: 52.0 months versus 34.7 months), although the survival analysis was not statistically significant. Besides, the high-risk group held higher enrichment scores for predicting radiotherapy response pathways. Therefore, combined radiotherapy may be a feasible treatment option, which needs to be verified in a future prospective study. Currently, multiple oncogenic signaling pathways have corresponding targeted drugs, which have been used in clinical practice. Our study observed that the JAK-STAT signaling pathway and VEGF signaling pathway were significantly enriched in the high-risk group. There was evidence suggesting that GBM-resident TAMs were polarized to an immunosuppressive phenotype by STAT3 activation [84, 85]. STAT3 activation blocks antigen presentation and T-cell activation by inhibiting the maturation of DC [86]. Targeting STAT3 also became an immune therapeutic strategy, which can modulate tumor-mediated immune suppression [87]. Studies showed that STAT3 signaling was associated with chemoresistance of TMZ in gliomas [88]. Therefore, JAK-STAT axis inhibitors could be an option for high-risk gliomas patients with *IDH* mutations. It is promising for high-risk patients to combine JAK-STAT targeting therapy with TMZ, radiotherapy, or immune-related therapy. Moreover, tumor growth and progression rely on oxygen and nutrients supplied by blood, and vascular endothelial growth factors (VEGF) play an essential role in angiogenesis and vascular permeability [89]. VEGF also can inhibit cytotoxic T-cell and DC development and promote the infiltration of immunosuppressive cells, inducing an immunosuppressive tumor microenvironment and promoting tumor growth by the immune escape of tumor [90]. In turn, immunosuppressive immune cells generate proangiogenic factors and improve angiogenesis, forming a positive feedback loop [91]. In addition, tumor cells produce VEGF-A, which can increase the expression level of PD-1, CTLA-4, and TIM-3 in CD8+ T cells, thereby inducing T-cell exhaustion [92]. In our study, the increased expression of VEGFA and VEGFC was in the high-risk group. It may be one of the reasons leading to the exhaustion of T cells in the high-risk group. Therefore, antibodies (Bevacizumab, Aflibercept, and Ramucirumab) or tyrosine kinase inhibitors (Sorafenib, Sunitinib, Regorafenib, and Pazopanib) targeting VEGF pathways could be effective in high-risk gliomas patients with *IDH* mutations. The application of anti-VEGF molecules could normalize tumor vessels, increasing immune cells infiltration and the delivery of chemotherapy drugs. It is a theoretical foundation for combined immunotherapy and chemotherapy in high-risk groups [91]. In sum, our results bring new treatment options for high-risk patients, but it still needs to be further verified in prospective trials.

There are also several limitations of the present study. Firstly, a limitation of this study is its retrospective nature. Although we had applied algorithms to evaluate the two groups (low- and high-risk) in predicting the sensitivity of immune checkpoint blockade therapy, more clinical data and further prospective studies are required. Secondly, an important clinical factor of LGGs, surgery resection margin, is not included in the study.

## 5. Conclusions

In summary, the six-gene immune-related prognostic signature is able to predict the outcome for patients with *IDH*-mutant LGGs independently. It is promising to be a biomarker to divide *IDH*-mutant LGGs patients into subgroups with distinct overall survival and immunophenotypes. Thus, the risk signature could be applied to personalized management and improve survival. High-risk patients with *IDH*-mutant LGGs may benefit from immunotherapy, radiotherapy, and targeted therapy, and using the risk signature can improve the clinical outcome of these patients. These results promoted the understanding of immune characteristics and clinical management and precise therapy of *IDH*-mutant LGGs.

## Data Availability

The RNA-seq data (Counts and FPKM) from the TCGA database were obtained by applying the “TCGAbiolinks” R package (https://bioconductor.org/packages/release/bioc/html/TCGAbiolinks.html). The RNA-seq data (FPKM) from the CGGA database were downloaded from the web (http://www.cgga.org.cn/download.jsp).

## Supplementary Materials

Supplementary Materials
*Figure S1.* Validation of the six-gene signature in *IDH*-mutant GBM patients. *Figure S2.* The expression levels of interleukin and interferons in the low- and high-risk group in the CGGA_693 cohort. *Figure S3.* Role of the six-gene signature in predicting therapeutic responses to radiotherapy in *IDH*-mutant LGGs. *Figure S4.* The infiltrating difference of 30 immune cells and stromal cells between the low- and high-risk groups in the TCGA and CGGA325 cohorts. *Figure S5.* The T-cell exhaustion status in *IDH*-mutant LGGs between low- and high-risk patients. *Figure S6.* The expression levels of immunomodulators molecule in the low- and high-risk groups. *Figure S7*. Univariate and multivariate Cox analyses showed that the risk signature was significantly correlated with OS in the CGGA_325 cohort. *Figure S8.* Distribution of 22 immune cells between low- and high-risk groups*. Table S1*. Exhausted T-cell-related gene list.
