# Supplementary material for: An Immune-Related Prognostic Signature for Predicting Clinical Outcomes and Immune Landscape in IDH-Mutant Lower-Grade Gliomas
Source: J Oncol. 2021 Dec 18;2021:3766685. doi: 10.1155/2021/3766685 (PMC8710162; doi:10.1155/2021/3766685)
Supplement: Supplementary Materials — Figure S1. Validation of the six-gene signature in IDH-mutant GBM patients. Figure S2. The expression levels of interleukin and interferons in the low- and high-risk group in the CGGA_693 cohort. Figure S3. Role of the six-gene signature in predicting therapeutic responses to radiotherapy in IDH-mutant LGGs. Figure S4. The infiltrating difference of 30 immune cells and stromal cells between the low- and high-risk groups in the TCGA and CGGA325 cohorts. Figure S5. The T-cell exhaustion status in IDH-mutant LGGs between low- and high-risk patients. Figure S6. The expression levels of immunomodulators molecule in the low- and high-risk groups. Figure S7. Univariate and multivariate Cox analyses showed that the risk signature was significantly correlated with OS in the CGGA_325 cohort. Figure S8. Distribution of 22 immune cells between low- and high-risk groups. Table S1. Exhausted T-cell-related gene list. [file Figure-S4.pdf › 3766685.f1/Figure-S4.pdf]

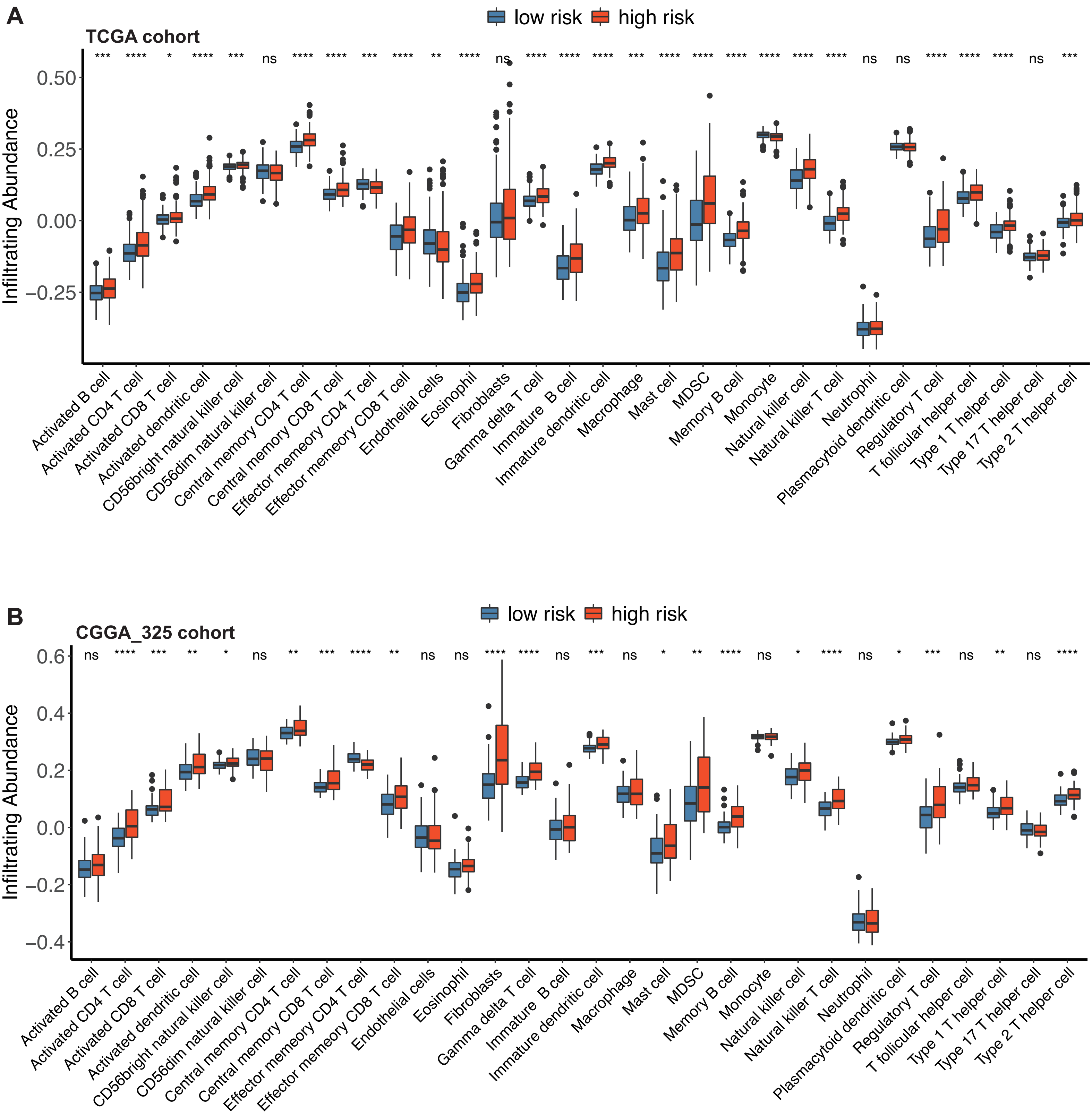

Figure S4. The infiltrating difference of 30 immune cells and stromal cells between the two groups in TCGA (**A**) and CGGA325 cohorts (**B**).
