# Supplementary material for: An Immune-Related Prognostic Signature for Predicting Clinical Outcomes and Immune Landscape in IDH-Mutant Lower-Grade Gliomas
Source: J Oncol. 2021 Dec 18;2021:3766685. doi: 10.1155/2021/3766685 (PMC8710162; doi:10.1155/2021/3766685)
Supplement: Supplementary Materials — Figure S1. Validation of the six-gene signature in IDH-mutant GBM patients. Figure S2. The expression levels of interleukin and interferons in the low- and high-risk group in the CGGA_693 cohort. Figure S3. Role of the six-gene signature in predicting therapeutic responses to radiotherapy in IDH-mutant LGGs. Figure S4. The infiltrating difference of 30 immune cells and stromal cells between the low- and high-risk groups in the TCGA and CGGA325 cohorts. Figure S5. The T-cell exhaustion status in IDH-mutant LGGs between low- and high-risk patients. Figure S6. The expression levels of immunomodulators molecule in the low- and high-risk groups. Figure S7. Univariate and multivariate Cox analyses showed that the risk signature was significantly correlated with OS in the CGGA_325 cohort. Figure S8. Distribution of 22 immune cells between low- and high-risk groups. Table S1. Exhausted T-cell-related gene list. [file 3766685.f1.zip › 3766685.f1/Table S1.pdf]

Table S1: Exhausted T cell related gene list.

Genes List
BATF
BTLA
CD160
CD244
CD274
CD276
CD40
CEACAM1
CTLA4
CXCR5
EOMES
FOXO1
FOXP3
GRB2
HAVCR2
ICOS
IDO1
IL10
IRF4
LAG3
MKI67
NFATC1
NFATC2IP
NFATC3
NFATC4
NFKB1
NFKB2
PDCD1
PLCG1
PLCG2
PRDM1
SATB1
TCF7
TGFB1

TIGIT
TNFRSF14
TNFRSF9
TOX
YY1
